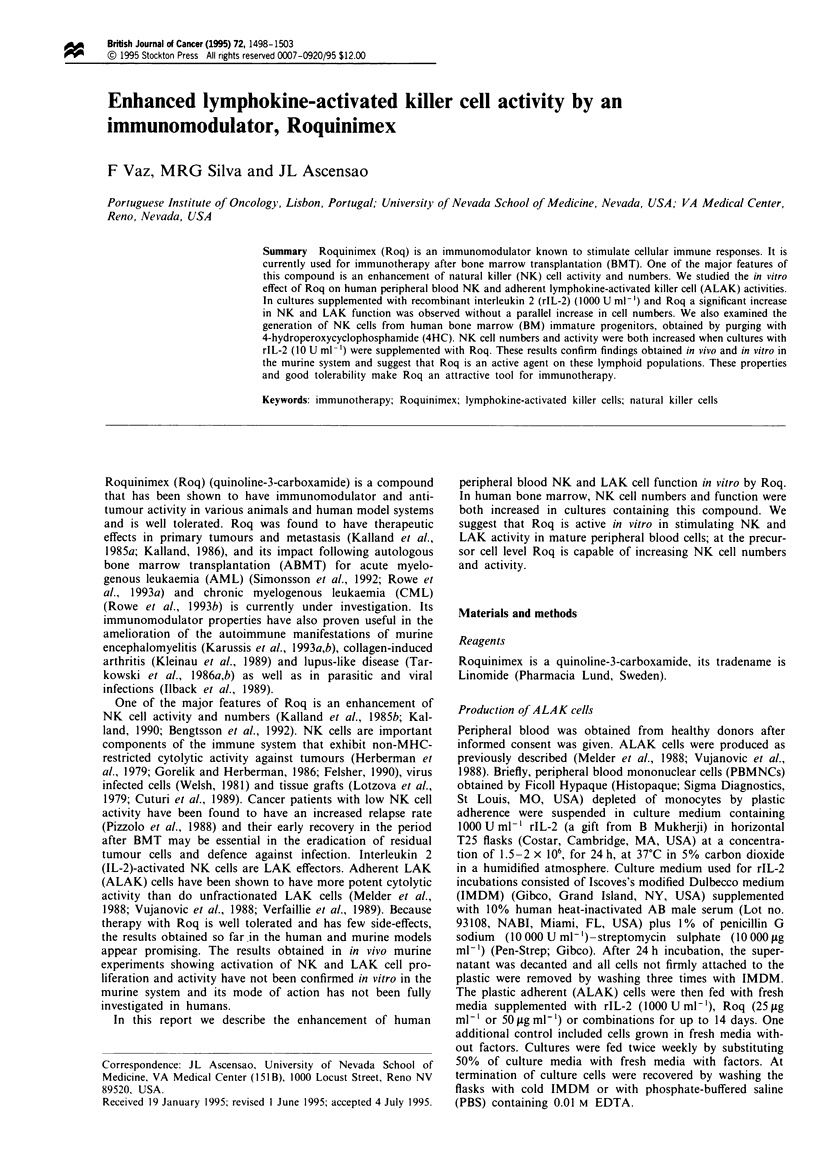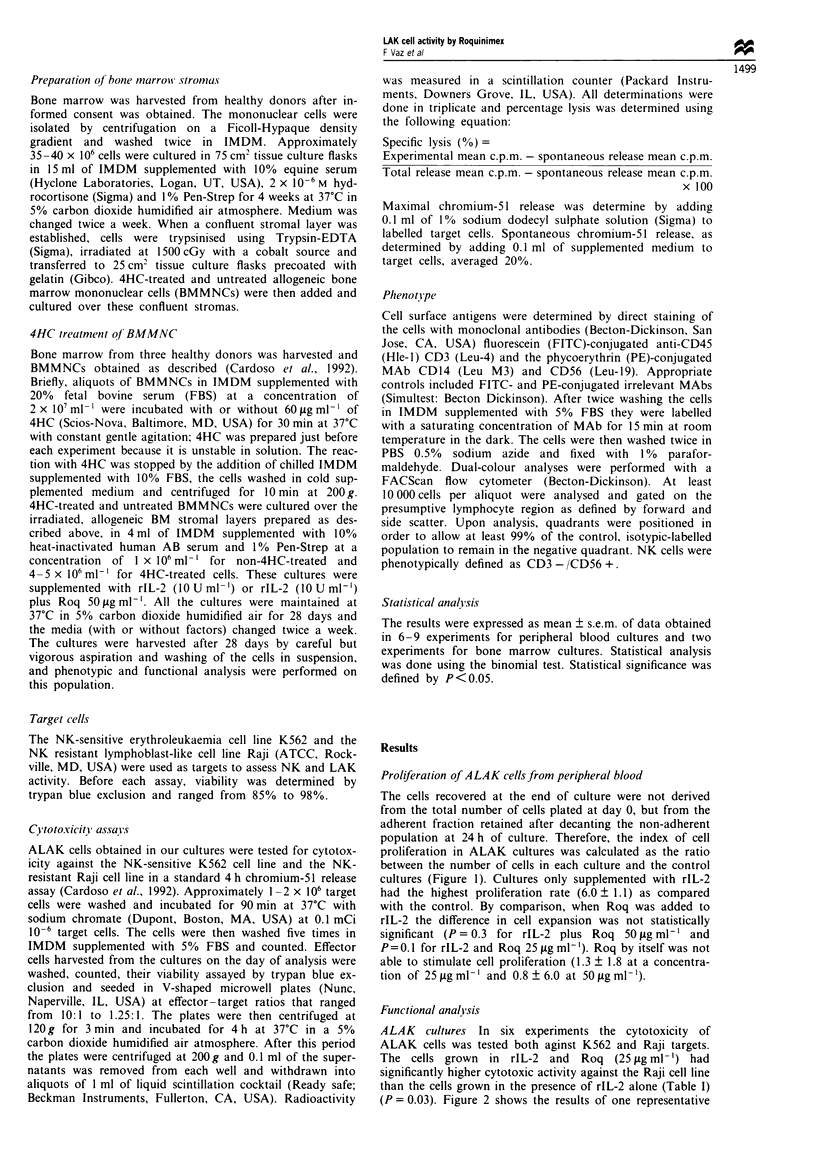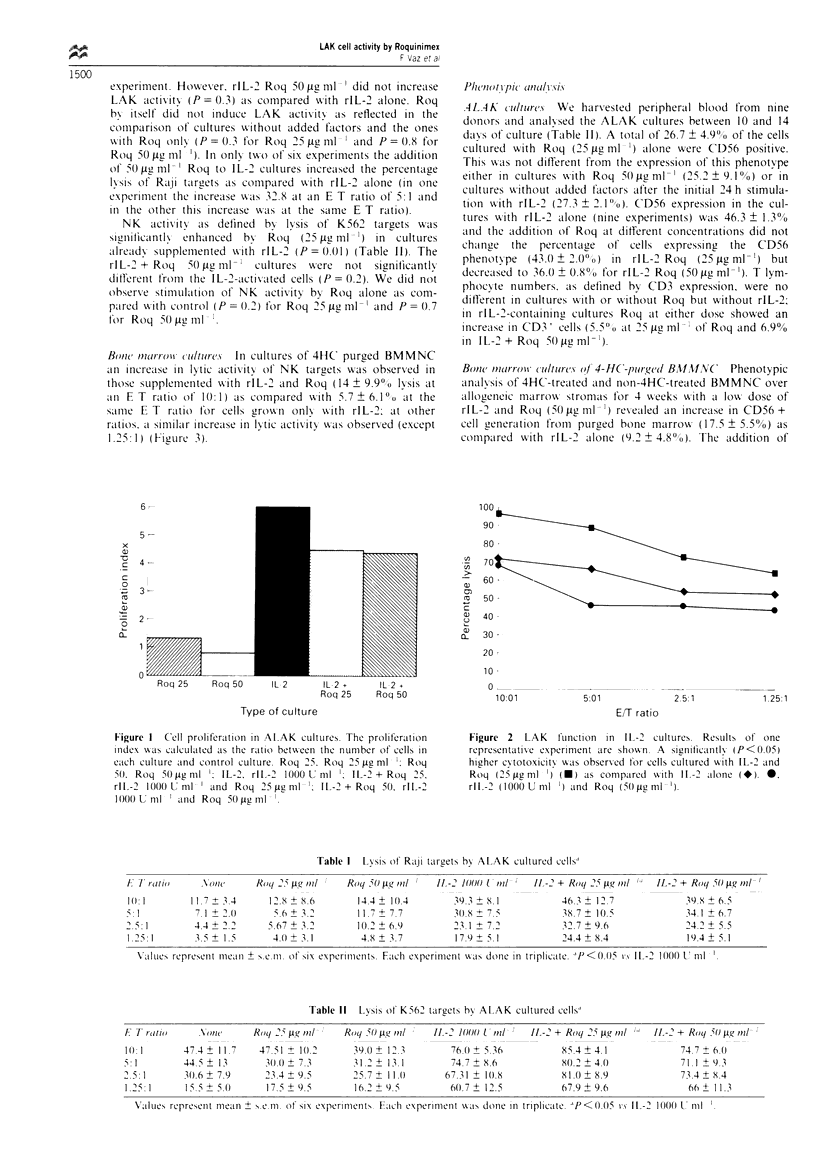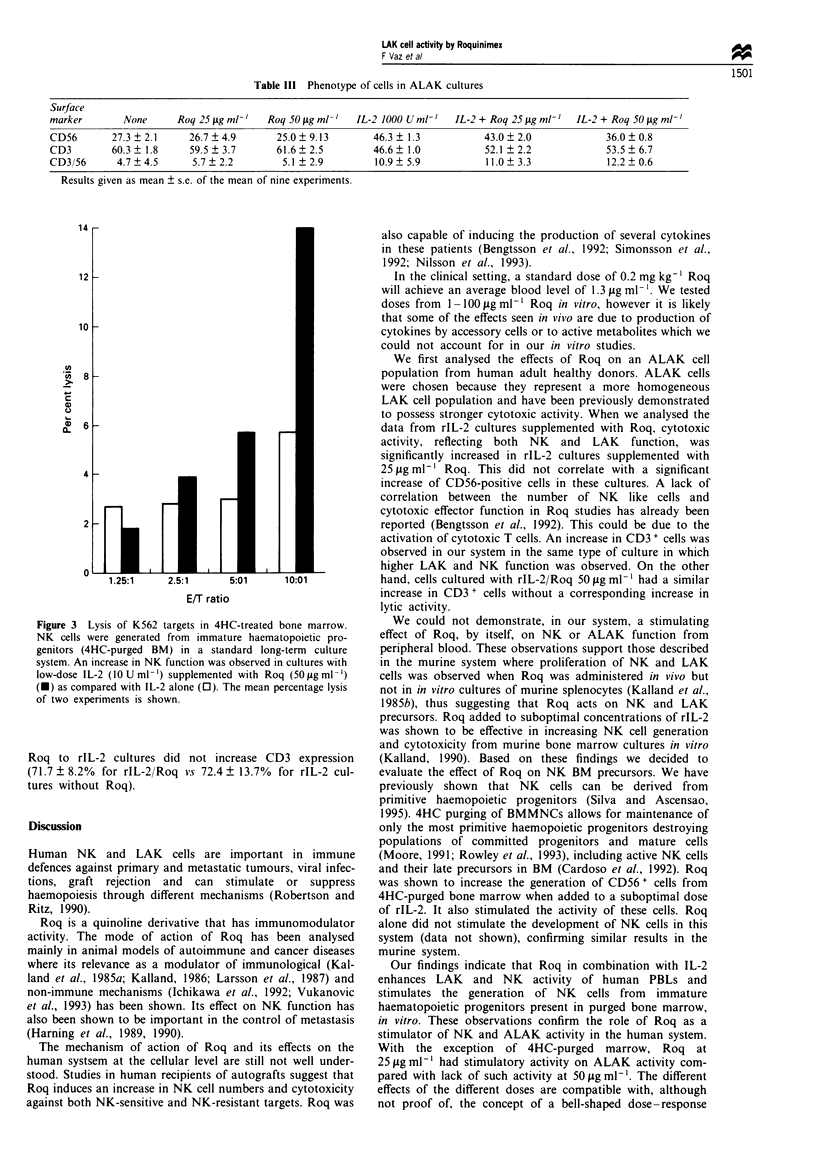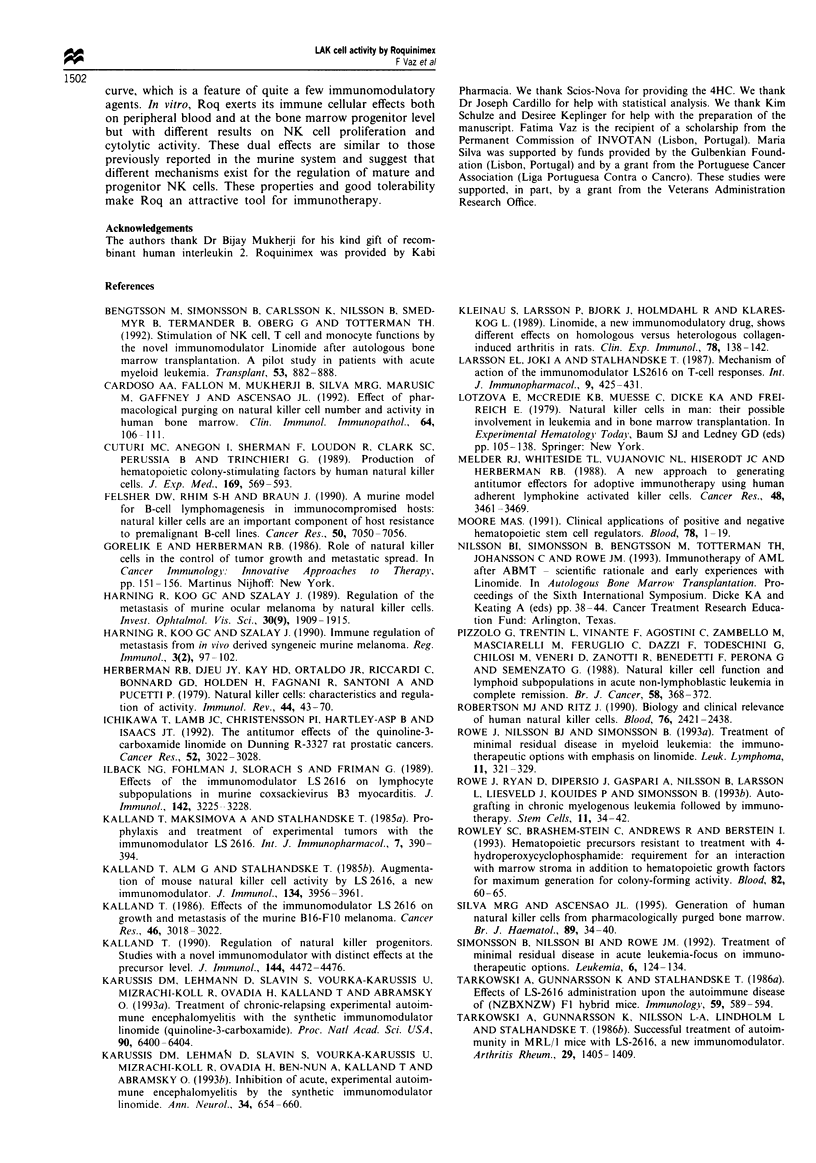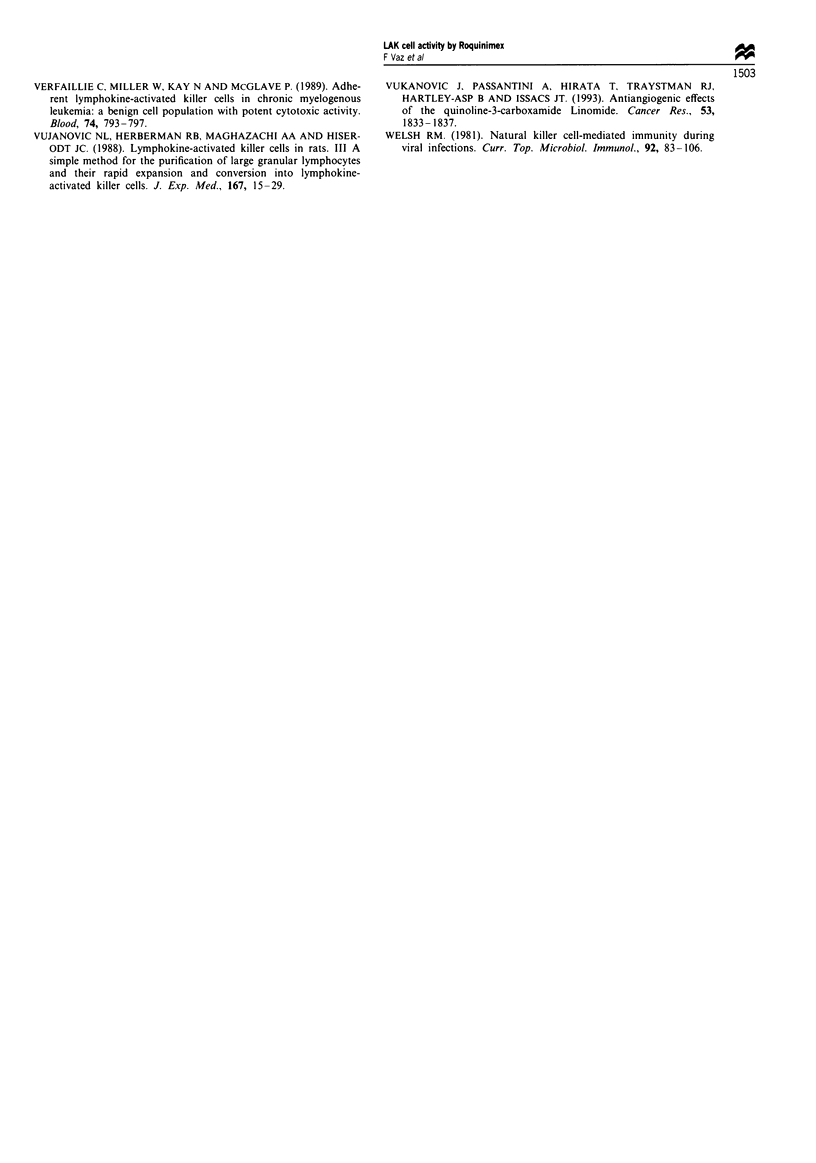# Enhanced lymphokine-activated killer cell activity by an immunomodulator, Roquinimex.

**DOI:** 10.1038/bjc.1995.536

**Published:** 1995-12

**Authors:** F. Vaz, M. R. Silva, J. L. Ascensao

**Affiliations:** Portuguese Institute of Oncology, Lisbon, Portugal.

## Abstract

Roquinimex (Roq) is an immunomodulator known to stimulate cellular immune responses. It is currently used for immunotherapy after bone marrow transplantation (BMT). One of the major features of this compound is an enhancement of natural killer (NK) cell activity and numbers. We studied the in vitro effect of Roq on human peripheral blood NK and adherent lymphokine-activated killer cell (ALAK) activities. In cultures supplemented with recombinant interleukin 2 (rIL-2) (1000 U ml-1) and Roq a significant increase in NK and LAK function was observed without a parallel increase in cell numbers. We also examined the generation of NK cells from human bone marrow (BM) immature progenitors, obtained by purging with 4-hydroperoxycyclophosphamide (4HC). NK cell numbers and activity were both increased when cultures with rIL-2 (10 U ml-1) were supplemented with Roq. These results confirm findings obtained in vivo and in vitro in the murine system and suggest that Roq is an active agent on these lymphoid populations. These properties and good tolerability make Roq an attractive tool for immunotherapy.


British Journal of Cancer (1995) 72, 1498-1503
© 1995 Stockton Press All rights reserved 0007-0920/95 $12.00

### Enhanced lymphokine-activated killer cell activity by an immunomodulator, Roquinimex

F Vaz, MRG Silva and JL Ascensao

*Portuguese Institute of Oncology, Lisbon, Portugal; University of Nevada School of Medicine, Nevada, USA; VA Medical Center, Reno, Nevada, USA*

**Summary** Roquinimex (Roq) is an immunomodulator known to stimulate cellular immune responses. It is currently used for immunotherapy after bone marrow transplantation (BMT). One of the major features of this compound is an enhancement of natural killer (NK) cell activity and numbers. We studied the *in vitro* effect of Roq on human peripheral blood NK and adherent lymphokine-activated killer cell (ALAK) activities. In cultures supplemented with recombinant interleukin 2 (rIL-2) (1000 U $ml^{-1}$) and Roq a significant increase in NK and LAK function was observed without a parallel increase in cell numbers. We also examined the generation of NK cells from human bone marrow (BM) immature progenitors, obtained by purging with 4-hydroperoxycyclophosphamide (4HC). NK cell numbers and activity were both increased when cultures with rIL-2 (10 U $ml^{-1}$) were supplemented with Roq. These results confirm findings obtained *in vivo* and *in vitro* in the murine system and suggest that Roq is an active agent on these lymphoid populations. These properties and good tolerability make Roq an attractive tool for immunotherapy.

**Keywords**: immunotherapy; Roquinimex; lymphokine-activated killer cells; natural killer cells

Roquinimex (Roq) (quinoline-3-carboxamide) is a compound that has been shown to have immunomodulator and anti-tumour activity in various animals and human model systems and is well tolerated. Roq was found to have therapeutic effects in primary tumours and metastasis (Kalland *et al.*, 1985*a*; Kalland, 1986), and its impact following autologous bone marrow transplantation (ABMT) for acute myelogenous leukaemia (AML) (Simonsson *et al.*, 1992; Rowe *et al.*, 1993*a*) and chronic myelogenous leukaemia (CML) (Rowe *et al.*, 1993*b*) is currently under investigation. Its immunomodulator properties have also proven useful in the amelioration of the autoimmune manifestations of murine encephalomyelitis (Karussis *et al.*, 1993*a*,*b*), collagen-induced arthritis (Kleinau *et al.*, 1989) and lupus-like disease (Tarkowski *et al.*, 1986*a*,*b*) as well as in parasitic and viral infections (Ilback *et al.*, 1989).

One of the major features of Roq is an enhancement of NK cell activity and numbers (Kalland *et al.*, 1985*b*; Kalland, 1990; Bengtsson *et al.*, 1992). NK cells are important components of the immune system that exhibit non-MHC-restricted cytolytic activity against tumours (Herberman *et al.*, 1979; Gorelik and Herberman, 1986; Felsher, 1990), virus infected cells (Welsh, 1981) and tissue grafts (Lotzova *et al.*, 1979; Cuturi *et al.*, 1989). Cancer patients with low NK cell activity have been found to have an increased relapse rate (Pizzolo *et al.*, 1988) and their early recovery in the period after BMT may be essential in the eradication of residual tumour cells and defence against infection. Interleukin 2 (IL-2)-activated NK cells are LAK effectors. Adherent LAK (ALAK) cells have been shown to have more potent cytolytic activity than do unfractionated LAK cells (Melder *et al.*, 1988; Vujanovic *et al.*, 1988; Verfaillie *et al.*, 1989). Because therapy with Roq is well tolerated and has few side-effects, the results obtained so far in the human and murine models appear promising. The results obtained in *in vivo* murine experiments showing activation of NK and LAK cell proliferation and activity have not been confirmed *in vitro* in the murine system and its mode of action has not been fully investigated in humans.

In this report we describe the enhancement of human peripheral blood NK and LAK cell function *in vitro* by Roq. In human bone marrow, NK cell numbers and function were both increased in cultures containing this compound. We suggest that Roq is active *in vitro* in stimulating NK and LAK activity in mature peripheral blood cells; at the precursor cell level Roq is capable of increasing NK cell numbers and activity.

Correspondence: JL Ascensao, University of Nevada School of Medicine, VA Medical Center (151B), 1000 Locust Street, Reno NV 89520, USA.
Received 19 January 1995; revised 1 June 1995; accepted 4 July 1995.

### Materials and methods

### Reagents

Roquinimex is a quinoline-3-carboxamide, its tradename is Linomide (Pharmacia Lund, Sweden).

### Production of ALAK cells

Peripheral blood was obtained from healthy donors after informed consent was given. ALAK cells were produced as previously described (Melder *et al.*, 1988; Vujanovic *et al.*, 1988). Briefly, peripheral blood mononuclear cells (PBMNCs) obtained by Ficoll Hypaque (Histopaque; Sigma Diagnostics, St Louis, MO, USA) depleted of monocytes by plastic adherence were suspended in culture medium containing 1000 U $ml^{-1}$ rIL-2 (a gift from B Mukherji) in horizontal T25 flasks (Costar, Cambridge, MA, USA) at a concentration of $1.5-2 \times 10^6$, for 24 h, at 37°C in 5% carbon dioxide in a humidified atmosphere. Culture medium used for rIL-2 incubations consisted of Iscoves's modified Dulbecco medium (IMDM) (Gibco, Grand Island, NY, USA) supplemented with 10% human heat-inactivated AB male serum (Lot no. 93108, NABI, Miami, FL, USA) plus 1% of penicillin G sodium (10 000 U $ml^{-1}$)-streptomycin sulphate (10 000 $\mu g$ $ml^{-1}$) (Pen-Strep; Gibco). After 24 h incubation, the supernatant was decanted and all cells not firmly attached to the plastic were removed by washing three times with IMDM. The plastic adherent (ALAK) cells were then fed with fresh media supplemented with rIL-2 (1000 U $ml^{-1}$), Roq (25 $\mu g$ $ml^{-1}$ or 50 $\mu g$ $ml^{-1}$) or combinations for up to 14 days. One additional control included cells grown in fresh media without factors. Cultures were fed twice weekly by substituting 50% of culture media with fresh media with factors. At termination of culture cells were recovered by washing the flasks with cold IMDM or with phosphate-buffered saline (PBS) containing 0.01 M EDTA.

*Preparation of bone marrow stromas*

Bone marrow was harvested from healthy donors after informed consent was obtained. The mononuclear cells were isolated by centrifugation on a Ficoll-Hypaque density gradient and washed twice in IMDM. Approximately 35–40 × $10^6$ cells were cultured in 75 $cm^2$ tissue culture flasks in 15 ml of IMDM supplemented with 10% equine serum (Hyclone Laboratories, Logan, UT, USA), 2 × $10^{-6}$ M hydrocortisone (Sigma) and 1% Pen-Strep for 4 weeks at 37°C in 5% carbon dioxide humidified air atmosphere. Medium was changed twice a week. When a confluent stromal layer was established, cells were trypsinised using Trypsin-EDTA (Sigma), irradiated at 1500 cGy with a cobalt source and transferred to 25 $cm^2$ tissue culture flasks precoated with gelatin (Gibco). 4HC-treated and untreated allogeneic bone marrow mononuclear cells (BMMNCs) were then added and cultured over these confluent stromas.

*4HC treatment of BMMNC*

Bone marrow from three healthy donors was harvested and BMMNCs obtained as described (Cardoso *et al.*, 1992). Briefly, aliquots of BMMNCs in IMDM supplemented with 20% fetal bovine serum (FBS) at a concentration of 2 × $10^7$ $ml^{-1}$ were incubated with or without 60 μg $ml^{-1}$ of 4HC (Scios-Nova, Baltimore, MD, USA) for 30 min at 37°C with constant gentle agitation; 4HC was prepared just before each experiment because it is unstable in solution. The reaction with 4HC was stopped by the addition of chilled IMDM supplemented with 10% FBS, the cells washed in cold supplemented medium and centrifuged for 10 min at 200 *g*. 4HC-treated and untreated BMMNCs were cultured over the irradiated, allogeneic BM stromal layers prepared as described above, in 4 ml of IMDM supplemented with 10% heat-inactivated human AB serum and 1% Pen-Strep at a concentration of 1 × $10^6$ $ml^{-1}$ for non-4HC-treated and 4–5 × $10^6$ $ml^{-1}$ for 4HC-treated cells. These cultures were supplemented with rIL-2 (10 U $ml^{-1}$) or rIL-2 (10 U $ml^{-1}$) plus Roq 50 μg $ml^{-1}$. All the cultures were maintained at 37°C in 5% carbon dioxide humidified air for 28 days and the media (with or without factors) changed twice a week. The cultures were harvested after 28 days by careful but vigorous aspiration and washing of the cells in suspension, and phenotypic and functional analysis were performed on this population.

*Target cells*

The NK-sensitive erythroleukaemia cell line K562 and the NK resistant lymphoblast-like cell line Raji (ATCC, Rockville, MD, USA) were used as targets to assess NK and LAK activity. Before each assay, viability was determined by trypan blue exclusion and ranged from 85% to 98%.

*Cytotoxicity assays*

ALAK cells obtained in our cultures were tested for cytotoxicity against the NK-sensitive K562 cell line and the NK-resistant Raji cell line in a standard 4 h chromium-51 release assay (Cardoso *et al.*, 1992). Approximately 1–2 × $10^6$ target cells were washed and incubated for 90 min at 37°C with sodium chromate (Dupont, Boston, MA, USA) at 0.1 mCi $10^{-6}$ target cells. The cells were then washed five times in IMDM supplemented with 5% FBS and counted. Effector cells harvested from the cultures on the day of analysis were washed, counted, their viability assayed by trypan blue exclusion and seeded in V-shaped microwell plates (Nunc, Naperville, IL, USA) at effector–target ratios that ranged from 10:1 to 1.25:1. The plates were then centrifuged at 120 *g* for 3 min and incubated for 4 h at 37°C in a 5% carbon dioxide humidified air atmosphere. After this period the plates were centrifuged at 200 *g* and 0.1 ml of the supernatants was removed from each well and withdrawn into aliquots of 1 ml of liquid scintillation cocktail (Ready safe; Beckman Instruments, Fullerton, CA, USA). Radioactivity was measured in a scintillation counter (Packard Instruments, Downers Grove, IL, USA). All determinations were done in triplicate and percentage lysis was determined using the following equation:

$$\text{Specific lysis (\%)} = \frac{\text{Experimental mean c.p.m.} - \text{spontaneous release mean c.p.m.}}{\text{Total release mean c.p.m.} - \text{spontaneous release mean c.p.m.}} \times 100$$

Maximal chromium-51 release was determine by adding 0.1 ml of 1% sodium dodecyl sulphate solution (Sigma) to labelled target cells. Spontaneous chromium-51 release, as determined by adding 0.1 ml of supplemented medium to target cells, averaged 20%.

*Phenotype*

Cell surface antigens were determined by direct staining of the cells with monoclonal antibodies (Becton-Dickinson, San Jose, CA, USA) fluorescein (FITC)-conjugated anti-CD45 (Hle-1) CD3 (Leu-4) and the phycoerythrin (PE)-conjugated MAb CD14 (Leu M3) and CD56 (Leu-19). Appropriate controls included FITC- and PE-conjugated irrelevant MAbs (Simultest: Becton Dickinson). After twice washing the cells in IMDM supplemented with 5% FBS they were labelled with a saturating concentration of MAb for 15 min at room temperature in the dark. The cells were then washed twice in PBS 0.5% sodium azide and fixed with 1% paraformaldehyde. Dual-colour analyses were performed with a FACScan flow cytometer (Becton-Dickinson). At least 10 000 cells per aliquot were analysed and gated on the presumptive lymphocyte region as defined by forward and side scatter. Upon analysis, quadrants were positioned in order to allow at least 99% of the control, isotypic-labelled population to remain in the negative quadrant. NK cells were phenotypically defined as CD3 –/CD56 +.

*Statistical analysis*

The results were expressed as mean ± s.e.m. of data obtained in 6–9 experiments for peripheral blood cultures and two experiments for bone marrow cultures. Statistical analysis was done using the binomial test. Statistical significance was defined by $P < 0.05$.

### Results

*Proliferation of ALAK cells from peripheral blood*

The cells recovered at the end of culture were not derived from the total number of cells plated at day 0, but from the adherent fraction retained after decanting the non-adherent population at 24 h of culture. Therefore, the index of cell proliferation in ALAK cultures was calculated as the ratio between the number of cells in each culture and the control cultures (Figure 1). Cultures only supplemented with rIL-2 had the highest proliferation rate (6.0 ± 1.1) as compared with the control. By comparison, when Roq was added to rIL-2 the difference in cell expansion was not statistically significant ($P = 0.3$ for rIL-2 plus Roq 50 μg $ml^{-1}$ and $P = 0.1$ for rIL-2 and Roq 25 μg $ml^{-1}$). Roq by itself was not able to stimulate cell proliferation (1.3 ± 1.8 at a concentration of 25 μg $ml^{-1}$ and 0.8 ± 6.0 at 50 μg $ml^{-1}$).

*Functional analysis*

*ALAK cultures* In six experiments the cytotoxicity of ALAK cells was tested both aginst K562 and Raji targets. The cells grown in rIL-2 and Roq (25 μg $ml^{-1}$) had significantly higher cytotoxic activity against the Raji cell line than the cells grown in the presence of rIL-2 alone (Table I) ($P = 0.03$). Figure 2 shows the results of one representative experiment. However, rIL-2 Roq 50 µg ml$^{-1}$ did not increase LAK activity ($P = 0.3$) as compared with rIL-2 alone. Roq by itself did not induce LAK activity as reflected in the comparison of cultures without added factors and the ones with Roq only ($P = 0.3$ for Roq 25 µg ml$^{-1}$ and $P = 0.8$ for Roq 50 µg ml$^{-1}$). In only two of six experiments the addition of 50 µg ml$^{-1}$ Roq to IL-2 cultures increased the percentage lysis of Raji targets as compared with rIL-2 alone (in one experiment the increase was 32.8 at an E T ratio of 5:1 and in the other this increase was at the same E T ratio).

NK activity as defined by lysis of K562 targets was significantly enhanced by Roq (25 µg ml$^{-1}$) in cultures already supplemented with rIL-2 ($P = 0.01$) (Table II). The rIL-2 + Roq 50 µg ml$^{-1}$ cultures were not significantly different from the IL-2-activated cells ($P = 0.2$). We did not observe stimulation of NK activity by Roq alone as compared with control ($P = 0.2$) for Roq 25 µg ml$^{-1}$ and $P = 0.7$ for Roq 50 µg ml$^{-1}$.

*Bone marrow cultures* In cultures of 4HC purged BMMNC an increase in lytic activity of NK targets was observed in those supplemented with rIL-2 and Roq (14 ± 9.9% lysis at an E T ratio of 10:1) as compared with 5.7 ± 6.1% at the same E T ratio for cells grown only with rIL-2; at other ratios, a similar increase in lytic activity was observed (except 1.25:1) (Figure 3).

*Phenotypic analysis*

*ALAK cultures* We harvested peripheral blood from nine donors and analysed the ALAK cultures between 10 and 14 days of culture (Table II). A total of 26.7 ± 4.9% of the cells cultured with Roq (25 µg ml$^{-1}$) alone were CD56 positive. This was not different from the expression of this phenotype either in cultures with Roq 50 µg ml$^{-1}$ (25.2 ± 9.1%) or in cultures without added factors after the initial 24 h stimulation with rIL-2 (27.3 ± 2.1%). CD56 expression in the cultures with rIL-2 alone (nine experiments) was 46.3 ± 1.3% and the addition of Roq at different concentrations did not change the percentage of cells expressing the CD56 phenotype (43.0 ± 2.0%) in rIL-2 Roq (25 µg ml$^{-1}$) but decreased to 36.0 ± 0.8% for rIL-2 Roq (50 µg ml$^{-1}$). T lymphocyte numbers, as defined by CD3 expression, were no different in cultures with or without Roq but without rIL-2; in rIL-2-containing cultures Roq at either dose showed an increase in CD3$^{+}$ cells (5.5% at 25 µg ml$^{-1}$ of Roq and 6.9% in IL-2 + Roq 50 µg ml$^{-1}$).

*Bone marrow cultures of 4-HC-purged BMMNC* Phenotypic analysis of 4HC-treated and non-4HC-treated BMMNC over allogeneic marrow stromas for 4 weeks with a low dose of rIL-2 and Roq (50 µg ml$^{-1}$) revealed an increase in CD56 + cell generation from purged bone marrow (17.5 ± 5.5%) as compared with rIL-2 alone (9.2 ± 4.8%). The addition of

**Figure 1** Cell proliferation in ALAK cultures. The proliferation index was calculated as the ratio between the number of cells in each culture and control culture. Roq 25, Roq 25 µg ml$^{-1}$; Roq 50, Roq 50 µg ml$^{-1}$; IL-2, rIL-2 1000 U ml$^{-1}$; IL-2 + Roq 25, rIL-2 1000 U ml$^{-1}$ and Roq 25 µg ml$^{-1}$; IL-2 + Roq 50, rIL-2 1000 U ml$^{-1}$ and Roq 50 µg ml$^{-1}$.

**Figure 2** LAK function in IL-2 cultures. Results of one representative experiment are shown. A significantly ($P < 0.05$) higher cytotoxicity was observed for cells cultured with IL-2 and Roq (25 µg ml$^{-1}$) (■) as compared with IL-2 alone (◆). ●, rIL-2 (1000 U ml$^{-1}$) and Roq (50 µg ml$^{-1}$).

**Table I** Lysis of Raji targets by ALAK cultured cells[a]

| *E T ratio* | *None* | *Roq 25 µg ml$^{-1}$* | *Roq 50 µg ml$^{-1}$* | *IL-2 1000 U ml$^{-1}$* | *IL-2 + Roq 25 µg ml$^{-1}$*[a] | *IL-2 + Roq 50 µg ml$^{-1}$* |
|---|---|---|---|---|---|---|
| 10:1 | 11.7 ± 3.4 | 12.8 ± 8.6 | 14.4 ± 10.4 | 39.3 ± 8.1 | 46.3 ± 12.7 | 39.8 ± 6.5 |
| 5:1 | 7.1 ± 2.0 | 5.6 ± 3.2 | 11.7 ± 7.7 | 30.8 ± 7.5 | 38.7 ± 10.5 | 34.1 ± 6.7 |
| 2.5:1 | 4.4 ± 2.2 | 5.67 ± 3.2 | 10.2 ± 6.9 | 23.1 ± 7.2 | 32.7 ± 9.6 | 24.2 ± 5.5 |
| 1.25:1 | 3.5 ± 1.5 | 4.0 ± 3.1 | 4.8 ± 3.7 | 17.9 ± 5.1 | 24.4 ± 8.4 | 19.4 ± 5.1 |

Values represent mean ± s.e.m. of six experiments. Each experiment was done in triplicate. [a]$P < 0.05$ *vs* IL-2 1000 U ml$^{-1}$.

**Table II** Lysis of K562 targets by ALAK cultured cells[a]

| *E T ratio* | *None* | *Roq 25 µg ml$^{-1}$* | *Roq 50 µg ml$^{-1}$* | *IL-2 1000 U ml$^{-1}$* | *IL-2 + Roq 25 µg ml$^{-1}$*[a] | *IL-2 + Roq 50 µg ml$^{-1}$* |
|---|---|---|---|---|---|---|
| 10:1 | 47.4 ± 11.7 | 47.51 ± 10.2 | 39.0 ± 12.3 | 76.0 ± 5.36 | 85.4 ± 4.1 | 74.7 ± 6.0 |
| 5:1 | 44.5 ± 13 | 30.0 ± 7.3 | 31.2 ± 13.1 | 74.7 ± 8.6 | 80.2 ± 4.0 | 71.1 ± 9.3 |
| 2.5:1 | 30.6 ± 7.9 | 23.4 ± 9.5 | 25.7 ± 11.0 | 67.31 ± 10.8 | 81.0 ± 8.9 | 73.4 ± 8.4 |
| 1.25:1 | 15.5 ± 5.0 | 17.5 ± 9.5 | 16.2 ± 9.5 | 60.7 ± 12.5 | 67.9 ± 9.6 | 66 ± 11.3 |

Values represent mean ± s.e.m. of six experiments. Each experiment was done in triplicate. [a]$P < 0.05$ *vs* IL-2 1000 U ml$^{-1}$.

**Table III** Phenotype of cells in ALAK cultures

| *Surface marker* | *None* | *Roq 25 μg ml⁻¹* | *Roq 50 μg ml⁻¹* | *IL-2 1000 U ml⁻¹* | *IL-2 + Roq 25 μg ml⁻¹* | *IL-2 + Roq 50 μg ml⁻¹* |
|---|---|---|---|---|---|---|
| CD56 | 27.3 ± 2.1 | 26.7 ± 4.9 | 25.0 ± 9.13 | 46.3 ± 1.3 | 43.0 ± 2.0 | 36.0 ± 0.8 |
| CD3 | 60.3 ± 1.8 | 59.5 ± 3.7 | 61.6 ± 2.5 | 46.6 ± 1.0 | 52.1 ± 2.2 | 53.5 ± 6.7 |
| CD3/56 | 4.7 ± 4.5 | 5.7 ± 2.2 | 5.1 ± 2.9 | 10.9 ± 5.9 | 11.0 ± 3.3 | 12.2 ± 0.6 |

Results given as mean ± s.e. of the mean of nine experiments.

**Figure 3** Lysis of K562 targets in 4HC-treated bone marrow. NK cells were generated from immature haematopoietic progenitors (4HC-purged BM) in a standard long-term culture system. An increase in NK function was observed in cultures with low-dose IL-2 (10 U $ml^{-1}$) supplemented with Roq (50 μg $ml^{-1}$) (■) as compared with IL-2 alone (□). The mean percentage lysis of two experiments is shown.

Roq to rIL-2 cultures did not increase CD3 expression (71.7 ± 8.2% for rIL-2/Roq *vs* 72.4 ± 13.7% for rIL-2 cultures without Roq).

### Discussion

Human NK and LAK cells are important in immune defences against primary and metastatic tumours, viral infections, graft rejection and can stimulate or suppress haemopoiesis through different mechanisms (Robertson and Ritz, 1990).

Roq is a quinoline derivative that has immunomodulator activity. The mode of action of Roq has been analysed mainly in animal models of autoimmune and cancer diseases where its relevance as a modulator of immunological (Kalland *et al.*, 1985*a*; Kalland, 1986; Larsson *et al.*, 1987) and non-immune mechanisms (Ichikawa *et al.*, 1992; Vukanovic *et al.*, 1993) has been shown. Its effect on NK function has also been shown to be important in the control of metastasis (Harning *et al.*, 1989, 1990).

The mechanism of action of Roq and its effects on the human systsem at the cellular level are still not well understood. Studies in human recipients of autografts suggest that Roq induces an increase in NK cell numbers and cytotoxicity against both NK-sensitive and NK-resistant targets. Roq was also capable of inducing the production of several cytokines in these patients (Bengtsson *et al.*, 1992; Simonsson *et al.*, 1992; Nilsson *et al.*, 1993).

In the clinical setting, a standard dose of 0.2 mg $kg^{-1}$ Roq will achieve an average blood level of 1.3 μg $ml^{-1}$. We tested doses from 1–100 μg $ml^{-1}$ Roq *in vitro*, however it is likely that some of the effects seen *in vivo* are due to production of cytokines by accessory cells or to active metabolites which we could not account for in our *in vitro* studies.

We first analysed the effects of Roq on an ALAK cell population from human adult healthy donors. ALAK cells were chosen because they represent a more homogeneous LAK cell population and have been previously demonstrated to possess stronger cytotoxic activity. When we analysed the data from rIL-2 cultures supplemented with Roq, cytotoxic activity, reflecting both NK and LAK function, was significantly increased in rIL-2 cultures supplemented with 25 μg $ml^{-1}$ Roq. This did not correlate with a significant increase of CD56-positive cells in these cultures. A lack of correlation between the number of NK like cells and cytotoxic effector function in Roq studies has already been reported (Bengtsson *et al.*, 1992). This could be due to the activation of cytotoxic T cells. An increase in $CD3^+$ cells was observed in our system in the same type of culture in which higher LAK and NK function was observed. On the other hand, cells cultured with rIL-2/Roq 50 μg $ml^{-1}$ had a similar increase in $CD3^+$ cells without a corresponding increase in lytic activity.

We could not demonstrate, in our system, a stimulating effect of Roq, by itself, on NK or ALAK function from peripheral blood. These observations support those described in the murine system where proliferation of NK and LAK cells was observed when Roq was administered *in vivo* but not in *in vitro* cultures of murine splenocytes (Kalland *et al.*, 1985*b*), thus suggesting that Roq acts on NK and LAK precursors. Roq added to suboptimal concentrations of rIL-2 was shown to be effective in increasing NK cell generation and cytotoxicity from murine bone marrow cultures *in vitro* (Kalland, 1990). Based on these findings we decided to evaluate the effect of Roq on NK BM precursors. We have previously shown that NK cells can be derived from primitive haemopoietic progenitors (Silva and Ascensao, 1995). 4HC purging of BMMNCs allows for maintenance of only the most primitive haemopoietic progenitors destroying populations of committed progenitors and mature cells (Moore, 1991; Rowley *et al.*, 1993), including active NK cells and their late precursors in BM (Cardoso *et al.*, 1992). Roq was shown to increase the generation of $CD56^+$ cells from 4HC-purged bone marrow when added to a suboptimal dose of rIL-2. It also stimulated the activity of these cells. Roq alone did not stimulate the development of NK cells in this system (data not shown), confirming similar results in the murine system.

Our findings indicate that Roq in combination with IL-2 enhances LAK and NK activity of human PBLs and stimulates the generation of NK cells from immature haematopoietic progenitors present in purged bone marrow, *in vitro*. These observations confirm the role of Roq as a stimulator of NK and ALAK activity in the human system. With the exception of 4HC-purged marrow, Roq at 25 μg $ml^{-1}$ had stimulatory activity on ALAK activity compared with lack of such activity at 50 μg $ml^{-1}$. The different effects of the different doses are compatible with, although not proof of, the concept of a bell-shaped dose–response curve, which is a feature of quite a few immunomodulatory agents. *In vitro*, Roq exerts its immune cellular effects both on peripheral blood and at the bone marrow progenitor level but with different results on NK cell proliferation and cytolytic activity. These dual effects are similar to those previously reported in the murine system and suggest that different mechanisms exist for the regulation of mature and progenitor NK cells. These properties and good tolerability make Roq an attractive tool for immunotherapy.

**Acknowledgements**
The authors thank Dr Bijay Mukherji for his kind gift of recombinant human interleukin 2. Roquinimex was provided by Kabi Pharmacia. We thank Scios-Nova for providing the 4HC. We thank Dr Joseph Cardillo for help with statistical analysis. We thank Kim Schulze and Desiree Keplinger for help with the preparation of the manuscript. Fatima Vaz is the recipient of a scholarship from the Permanent Commission of INVOTAN (Lisbon, Portugal). Maria Silva was supported by funds provided by the Gulbenkian Foundation (Lisbon, Portugal) and by a grant from the Portuguese Cancer Association (Liga Portuguesa Contra o Cancro). These studies were supported, in part, by a grant from the Veterans Administration Research Office.

**References**

BENGTSSON M, SIMONSSON B, CARLSSON K, NILSSON B, SMEDMYR B, TERMANDER B, OBERG G AND TOTTERMAN TH. (1992). Stimulation of NK cell, T cell and monocyte functions by the novel immunomodulator Linomide after autologous bone marrow transplantation. A pilot study in patients with acute myeloid leukemia. *Transplant*, **53**, 882–888.

CARDOSO AA, FALLON M, MUKHERJI B, SILVA MRG, MARUSIC M, GAFFNEY J AND ASCENSAO JL. (1992). Effect of pharmacological purging on natural killer cell number and activity in human bone marrow. *Clin. Immunol. Immunopathol.*, **64**, 106–111.

CUTURI MC, ANEGON I, SHERMAN F, LOUDON R, CLARK SC, PERUSSIA B AND TRINCHIERI G. (1989). Production of hematopoietic colony-stimulating factors by human natural killer cells. *J. Exp. Med.*, **169**, 569–593.

FELSHER DW, RHIM S-H AND BRAUN J. (1990). A murine model for B-cell lymphomagenesis in immunocompromised hosts: natural killer cells are an important component of host resistance to premalignant B-cell lines. *Cancer Res.*, **50**, 7050–7056.

GORELIK E AND HERBERMAN RB. (1986). Role of natural killer cells in the control of tumor growth and metastatic spread. In *Cancer Immunology: Innovative Approaches to Therapy*, pp. 151–156. Martinus Nijhoff: New York.

HARNING R, KOO GC AND SZALAY J. (1989). Regulation of the metastasis of murine ocular melanoma by natural killer cells. *Invest. Ophtalmol. Vis. Sci.*, **30(9)**, 1909–1915.

HARNING R, KOO GC AND SZALAY J. (1990). Immune regulation of metastasis from *in vivo* derived syngeneic murine melanoma. *Reg. Immunol.*, **3(2)**, 97–102.

HERBERMAN RB, DJEU JY, KAY HD, ORTALDO JR, RICCARDI C, BONNARD GD, HOLDEN H, FAGNANI R, SANTONI A AND PUCETTI P. (1979). Natural killer cells: characteristics and regulation of activity. *Immunol. Rev.*, **44**, 43–70.

ICHIKAWA T, LAMB JC, CHRISTENSSON PI, HARTLEY-ASP B AND ISAACS JT. (1992). The antitumor effects of the quinoline-3-carboxamide linomide on Dunning R-3327 rat prostatic cancers. *Cancer Res.*, **52**, 3022–3028.

ILBACK NG, FOHLMAN J, SLORACH S AND FRIMAN G. (1989). Effects of the immunomodulator LS 2616 on lymphocyte subpopulations in murine coxsackievirus B3 myocarditis. *J. Immunol.*, **142**, 3225–3228.

KALLAND T, MAKSIMOVA A AND STALHANDSKE T. (1985*a*). Prophylaxis and treatment of experimental tumors with the immunomodulator LS 2616. *Int. J. Immunopharmacol.*, **7**, 390–394.

KALLAND T, ALM G AND STALHANDSKE T. (1985*b*). Augmentation of mouse natural killer cell activity by LS 2616, a new immunomodulator. *J. Immunol.*, **134**, 3956–3961.

KALLAND T. (1986). Effects of the immunomodulator LS 2616 on growth and metastasis of the murine B16-F10 melanoma. *Cancer Res.*, **46**, 3018–3022.

KALLAND T. (1990). Regulation of natural killer progenitors. Studies with a novel immunomodulator with distinct effects at the precursor level. *J. Immunol.*, **144**, 4472–4476.

KARUSSIS DM, LEHMANN D, SLAVIN S, VOURKA-KARUSSIS U, MIZRACHI-KOLL R, OVADIA H, KALLAND T AND ABRAMSKY O. (1993*a*). Treatment of chronic-relapsing experimental autoimmune encephalomyelitis with the synthetic immunomodulator linomide (quinoline-3-carboxamide). *Proc. Natl Acad. Sci. USA*, **90**, 6400–6404.

KARUSSIS DM, LEHMAN D, SLAVIN S, VOURKA-KARUSSIS U, MIZRACHI-KOLL R, OVADIA H, BEN-NUN A, KALLAND T AND ABRAMSKY O. (1993*b*). Inhibition of acute, experimental autoimmune encephalomyelitis by the synthetic immunomodulator linomide. *Ann. Neurol.*, **34**, 654–660.

KLEINAU S, LARSSON P, BJORK J, HOLMDAHL R AND KLARESKOG L. (1989). Linomide, a new immunomodulatory drug, shows different effects on homologous versus heterologous collagen-induced arthritis in rats. *Clin. Exp. Immunol.*, **78**, 138–142.

LARSSON EL, JOKI A AND STALHANDSKE T. (1987). Mechanism of action of the immunomodulator LS2616 on T-cell responses. *Int. J. Immunopharmacol.*, **9**, 425–431.

LOTZOVA E, MCCREDIE KB, MUESSE C, DICKE KA AND FREIREICH E. (1979). Natural killer cells in man: their possible involvement in leukemia and in bone marrow transplantation. In *Experimental Hematology Today*, Baum SJ and Ledney GD (eds) pp. 105–138. Springer: New York.

MELDER RJ, WHITESIDE TL, VUJANOVIC NL, HISERODT JC AND HERBERMAN RB. (1988). A new approach to generating antitumor effectors for adoptive immunotherapy using human adherent lymphokine activated killer cells. *Cancer Res.*, **48**, 3461–3469.

MOORE MAS. (1991). Clinical applications of positive and negative hematopoietic stem cell regulators. *Blood*, **78**, 1–19.

NILSSON BI, SIMONSSON B, BENGTSSON M, TOTTERMAN TH, JOHANSSON C AND ROWE JM. (1993). Immunotherapy of AML after ABMT – scientific rationale and early experiences with Linomide. In *Autologous Bone Marrow Transplantation*. Proceedings of the Sixth International Symposium. Dicke KA and Keating A (eds) pp. 38–44. Cancer Treatment Research Education Fund: Arlington, Texas.

PIZZOLO G, TRENTIN L, VINANTE F, AGOSTINI C, ZAMBELLO M, MASCIARELLI M, FERUGLIO C, DAZZI F, TODESCHINI G, CHILOSI M, VENERI D, ZANOTTI R, BENEDETTI F, PERONA G AND SEMENZATO G. (1988). Natural killer cell function and lymphoid subpopulations in acute non-lymphoblastic leukemia in complete remission. *Br. J. Cancer*, **58**, 368–372.

ROBERTSON MJ AND RITZ J. (1990). Biology and clinical relevance of human natural killer cells. *Blood*, **76**, 2421–2438.

ROWE J, NILSSON BJ AND SIMONSSON B. (1993*a*). Treatment of minimal residual disease in myeloid leukemia: the immunotherapeutic options with emphasis on linomide. *Leuk. Lymphoma*, **11**, 321–329.

ROWE J, RYAN D, DIPERSIO J, GASPARI A, NILSSON B, LARSSON L, LIESVELD J, KOUIDES P AND SIMONSSON B. (1993*b*). Autografting in chronic myelogenous leukemia followed by immunotherapy. *Stem Cells*, **11**, 34–42.

ROWLEY SC, BRASHEM-STEIN C, ANDREWS R AND BERSTEIN I. (1993). Hematopoietic precursors resistant to treatment with 4-hydroperoxycyclophosphamide: requirement for an interaction with marrow stroma in addition to hematopoietic growth factors for maximum generation for colony-forming activity. *Blood*, **82**, 60–65.

SILVA MRG AND ASCENSAO JL. (1995). Generation of human natural killer cells from pharmacologically purged bone marrow. *Br. J. Haematol.*, **89**, 34–40.

SIMONSSON B, NILSSON BI AND ROWE JM. (1992). Treatment of minimal residual disease in acute leukemia-focus on immunotherapeutic options. *Leukemia*, **6**, 124–134.

TARKOWSKI A, GUNNARSSON K AND STALHANDSKE T. (1986*a*). Effects of LS-2616 administration upon the autoimmune disease of (NZBXNZW) F1 hybrid mice. *Immunology*, **59**, 589–594.

TARKOWSKI A, GUNNARSSON K, NILSSON L-A, LINDHOLM L AND STALHANDSKE T. (1986*b*). Successful treatment of autoimmunity in MRL/1 mice with LS-2616, a new immunomodulator. *Arthritis Rheum.*, **29**, 1405–1409.

VERFAILLIE C, MILLER W, KAY N AND MCGLAVE P. (1989). Adherent lymphokine-activated killer cells in chronic myelogenous leukemia: a benign cell population with potent cytotoxic activity. *Blood*, **74,** 793-797.

VUJANOVIC NL, HERBERMAN RB, MAGHAZACHI AA AND HISERODT JC. (1988). Lymphokine-activated killer cells in rats. III A simple method for the purification of large granular lymphocytes and their rapid expansion and conversion into lymphokine-activated killer cells. *J. Exp. Med.*, **167,** 15-29.

VUKANOVIC J, PASSANTINI A, HIRATA T, TRAYSTMAN RJ, HARTLEY-ASP B AND ISSACS JT. (1993). Antiangiogenic effects of the quinoline-3-carboxamide Linomide. *Cancer Res.*, **53,** 1833-1837.

WELSH RM. (1981). Natural killer cell-mediated immunity during viral infections. *Curr. Top. Microbiol. Immunol.*, **92,** 83-106.